# Analyses of air pollution control measures and co-benefits in the heavily air-polluted Jinan city of China, 2013–2017

**DOI:** 10.1038/s41598-020-62475-0

**Published:** 2020-03-25

**Authors:** Liangliang Cui, Jingwen Zhou, Xiumiao Peng, Shiman Ruan, Ying Zhang

**Affiliations:** 1 Department of Environmental Health, Jinan Municipal Center for Disease Control and Prevention, Jinan City, Shandong Province 250021 China; 2 School of Public Health, University of Sydney, NSW 2006 Sydney, Australia

**Keywords:** Environmental impact, Environmental economics, Risk factors

## Abstract

China has made great efforts in air pollution control since 2013. However, there is a lack of evaluation of environmental, health and economic co-benefits associated with the national and local air pollution control measures at a city level. We analyzed local air pollution control policies and implementation in Jinan, one of the most heavily air-polluted cities in China between 2013 and 2017. We assessed the changes in exhaust emissions, air quality, mortality and morbidity of associated specific-diseases, and related economic benefits. We also projected the future scenarios of PM_2.5_ concentration dropped to 15 μg/m^3^. There were significant decreases in exhaust emissions of SO_2_ and NO_x_ in Jinan during the study period. Annual reductions in ambient air pollution were 72.6% for SO_2_, 43.1% for PM_2.5_, and 34.2% for PM_10_. A total of 2,317 (95%CI: 1,533–2,842) premature deaths and 15,822 (95%CI: 8,734–23,990) related morbidity cases had been avoided in 2017, leading to a total of US$ 317.7 million (95%CI: 227.5–458.1) in economic benefits. Decreasing PM_2.5_ concentrations to 15 μg/m^3^ would result in reductions of 70% in total PM_2.5_-related non-accidental mortality and 95% in total PM_2.5_-related morbidity, which translates into US$ 1,289.5 million (95%CI: 825.8–1,673.6) in economic benefits. The national and local air pollution control measures have brought significant environmental, health and economic benefits to a previously heavy polluted Chinese city.

## Introduction

Air pollution is a major global health concern, especially in developing countries like China. In January 2013, a severe air pollution event affected one-third of the major cities in China^1^. Since then, air pollution, especially the risks from PM_2.5_ (particle matter with aerodynamic diameter ≤ 2.5 μm), has become a growing concern in China^2,3^. Spatial and temporal distributions of air quality in China show that winter months in northern cities are the most polluted because of coal burning and other fossil fuel combustion for heating^4–7^.

Scientific studies have provided strong evidence on the effects of PM_2.5_ on mortality and morbidity, and for both short-term and long-term exposures^8,9^. In May 2018, the World Health Organization (WHO) reported that 4.2 million deaths occur annually as a result of exposure to ambient air pollution globally with developing countries having the highest burden^10^. The first WHO Global Conference on Air Pollution and Health held in November 2018 emphasized an aspirational goal of reducing the number of deaths from air pollution by two-thirds by 2030 and the urgent need for bold and prompt actions to address the present health crisis caused by air pollution^11^.

In order to improve air quality and to protect human health, the Chinese government has implemented a series of national policies to reduce the emissions of air pollutants after the 2013 national severe air pollution event^4,12^. In September 2013, the State Council of China issued the Air Pollution Prevention and Control Action Plan (APPCAP), which comprises ten specific tasks (Ten Tasks) with specific concentration targets to be achieved by 2017 in order to reduce air pollution^13^. For example, by 2017, urban concentrations of PM_10_ shall be reduced by 10% compared with that in 2012; PM_2.5_ concentrations for Beijing-Tianjin-Hebei region shall be lowered by 25%; 20% reduction in the Yangtze River Delta region and 15% reduction in the Pearl River Delta region compared with that in 2013.

Recent studies have demonstrated achieved health and economic benefits from the national APPCAP^4,14–16^. A clear reduction in air pollutants has been shown in many cities and regions^4,14,17,18^. Associated mortality and related economic benefits have also been reported due to improved air quality^13,18,19^. Wang *et al*.^4^ reviewed the effect of PM_2.5_ control measures and found a 24% reduction in PM_2.5_ during 2013 and 2015 in 74 major cities of China. Feng *et al*.^18^ found more than seventy percent of the studied Chinese cities achieved reduced PM_2.5_ from 2015 to 2016, and avoided a total of 10,658 per million deaths. Huang *et al*.^14^ reported that annual average concentrations of PM_2.5_ decreased by 33.3% in 74 Chinese cities between 2013 and 2017, and 47,240 fewer deaths due to the reduction. Chen *et al*.^17^ projected the mortality and economic benefits from a 25% reduction in PM_2.5_ concentration below the baseline of 2012 to 2014 in Beijing-Tianjin-Hebei region. Gao *et al*.^18^ conducted a cost-benefit analysis of data from 31 provinces and found a total of US$ 112.6 billion saved due to reduced mortality during the national APPCAP implementation. However, there was a lack of estimation on the impacts on morbidity and corresponding economic benefits attributable to reduction in PM_2.5_ concentration. In order to have a better understanding of the co-benefits that occurred during the national APPCAP implementation, a comprehensive evaluation of the improvement in air quality, benefits for both mortality and morbidity, and related economic benefits could aid policy making for air pollution control.

Situations of air pollution in China vary across regions and cities due to different natural and climate conditions, topography, sources of air pollution, and energy combustions^4,14,17,20^. In addition to national policies, localized measures are required in responding to specific air pollutants. Therefore, there is a need to examine city-level experience to reveal how local measures could benefit environment, health and economy, in addition to the national APPCAP. In particular, there is a limited understanding of what air pollution control measures have been implemented at a local level and their effectiveness and benefits.

Jinan is one of the most heavily air-polluted cities in China with a PM_2.5_ historical record of 443 ug/m^3^ during the severe air pollution event in 2013^21^. We selected Jinan city as the study site to quantify the co-benefits of local air pollution control measures and implementation between 2013 and 2017. The co-benefits estimated in this study focused on environmental, health and economic benefits from air pollution policies and measures. Previous local epidemiological studies have demonstrated the impacts of air pollution on mortality^22,23^ and morbidity^1,21,24^. Our findings will assist policy making and development to improve air quality and achieve health and economic benefits at local and national levels.

## Methods

**Figure 1:**
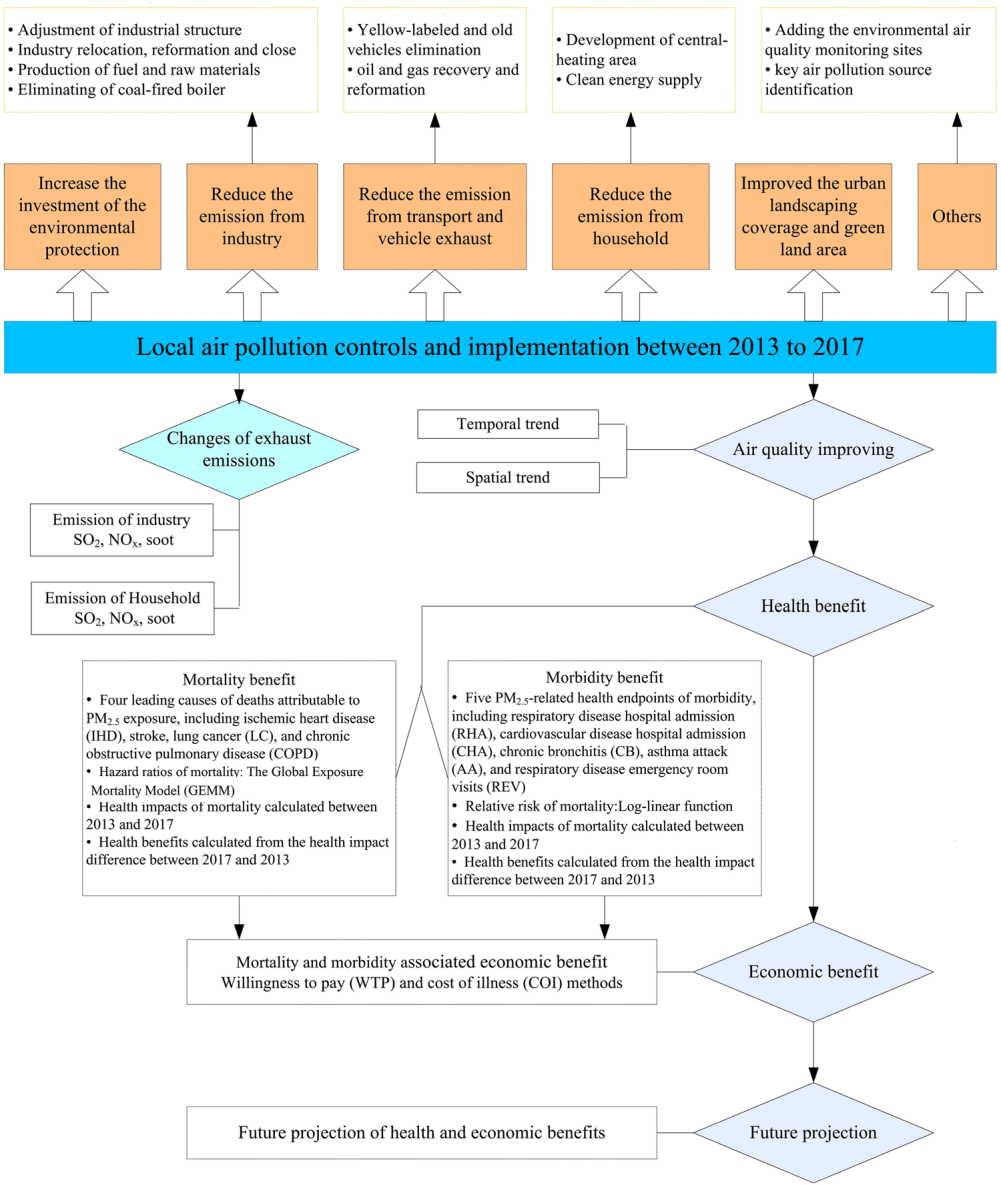
Methodological framework of the study.

The methodological framework of our study was shown in Fig. 1. We firstly reviewed air pollution control policies and measures between 2013 and 2017. We assessed the changes in exhaust emissions and ambient air pollutants during the study period. The reduction in mortality and morbidity from improved air pollution were quantified. Finally, associated economic benefit was estimated in US dollars. We further projected the future benefits with a scenario of PM_2.5_ concentration down to the national ambient air quality standards Grade-II (15 μg/m^3^)^25^.

### Background information for Jinan

**Figure 2:**
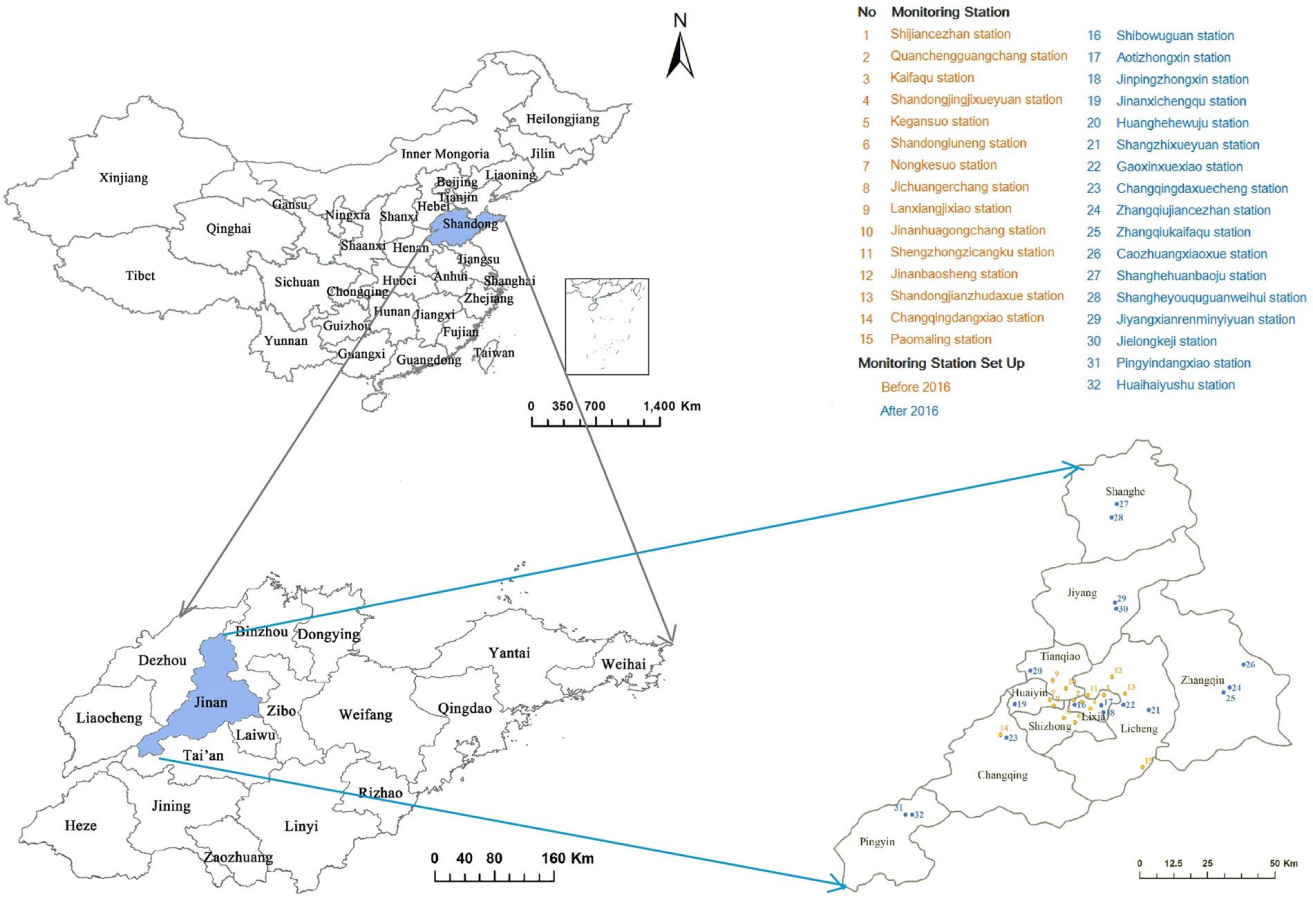
Jinan map and location of air pollution monitoring stations. Map was performed using R software (version 3.2.2, https://mirrors.tuna.tsinghua.edu.cn/CRAN/). The packages of *mapdata* (https://cran.r-project.org/web/packages/mapdata/index.html), *maps* (https://cran.r-project.org/web/packages/maps/index.html) and *ggmap* (https://cran.r-project.org/web/packages/ggmap/) were applied.

Jinan, the capital of Shandong province, comprises a total of 8,177.21 km^2^ with ten districts. It is located at 36.40°N latitude and 110.00°E longitude, along the coast of the Yellow River, downstream of the Central China Region (Fig. 2). It has become the core connection center for two economic zones: Beijing-Tianjin-Hebei Regions and Yangtze River Delta. In the past few years, Jinan has experienced rapid industrialisation and urbanisation. Nearly 40% of the gross domestic product (GDP) in Jinan was contributed by typical industries, including power plant, machinery manufacture, textile and steel production, chemical manufacturing, light industry, and building materials^21^. The GDP in Jinan was US$108.4 billion in 2017, increased by 37.70% from 2013 (US$78.7 billion). It has a stable population growth from 7.0 million in 2013 to 7.3 million in 2017, with nearly 15% of older population (65 years and older)^26^ (Fig. S1).

### Air pollution control measures

In responding to the national APPCAP and Ten Tasks, Jinan government released the Jinan Air Pollution Prevention and Control Action Plan (phase I) on 12 December 2013^27^ and Jinan Air Pollution Prevention and Control Action Plan (phase II) on 21 October 2016^28^, in which specific goals and detailed control measures were introduced to achieve the national goals set in the APPCAP. Jinan has also developed a range of local policies and measures to reduce air pollutants emissions in the last few years^29^. We reviewed all air pollution control policies in Jinan between 2013 and 2017, including documents from Jinan Environmental Protection Bureau^29^, Jinan Statistical Yearbook^26^, Shandong Statistical Yearbook^30–34^, and National Statistical Reports^35^. We also collected the air pollution control measures from the transportation sector from the Yellow-labeled vehicles (the government put labels on vehicles that don’t meet exhaust emission standards) and elimination policy, exhaust limitation, and oil reformation for old vehicles.

### Exhaust emissions

The exhaust of sulphur dioxide (SO_2_), nitrogen oxides (NO_x_), and soot from industries and households were extracted from Jinan Statistical Yearbook^26^.

### Air pollution data

Daily mean air pollutant concentration data of inhalable PM_2.5_, PM_10_, SO_2_, NO_2_ and CO, and daily maximum of 8-hourly running mean of O_3_ were obtained from Jinan Environmental Monitoring Center from January 1, 2013 to December 31, 2017. The data of 2013–2015 were obtained from 15 fixed air monitoring stations, and data of 2016–2017 were obtained from 32 fixed air monitoring stations (Fig. 2). The annual average concentration of air pollutants in city and districts were calculated by the mean of air pollutants concentration in the fixed air monitoring stations. Before 2016, four districts (Shanghe, Jiyang, Zhangqiu, and Pingyin) had no air monitoring station. Therefore the annual average concentration of air pollutants in the four districts between 2013 and 2015 were calculated by the average of 15 fixed air monitoring stations.

### Mortality and morbidity data

Four leading causes of mortality attributable to PM_2.5_ exposure, including ischemic heart disease (IHD), stroke, lung cancer (LC), and chronic obstructive pulmonary disease (COPD), recommended by Global Burden of Disease (GBD) study were included^3^. We obtained the four cause-specific mortalities (age > 25 years) of Jinan between 2013 and 2017 from Jinan Municipal Center for Disease Control and Prevention, which is responsible for death surveillance.

We selected five PM_2.5_-related health endpoints of morbidity, including respiratory disease hospital admission (RHA), cardiovascular disease hospital admission (CHA), chronic bronchitis (CB), asthma attack (AA), and respiratory disease emergency room visits (REV). Because of limited local data for the cause-specific morbidities, we used the national cause-specific morbidity rate of RHA, CHA, CB, AA, and REV as the reference morbidity in our estimation, which were collected from China Statistical Yearbook^36^.

### Estimation of health effect

We firstly estimated the long-term burden of mortality and morbidity attributable to PM_2.5_ in Jinan in 2013 and 2017, and then calculated the reduction in mortality and morbidity between 2013 and 2017.

#### Hazard ratios of mortality

The Global Exposure Mortality Model (GEMM) constructed by Burnett *et al*. 2018^37^ was used to examine the long-term mortality effects between 2013 and 2017. GEMM modeled the hazard ratio association between outdoor PM_2.5_ and non-accidental mortality using data from 41 cohorts of 16 countries, including the Chinese Male Cohort. GEMM for each of the five specific causes of GEMM 5-COD was used to estimate the combined population-attributable fraction based on five specific causes of death (age > 25 years) examined by the GBD. In our study, we estimated the IHD, stroke, LC, and COPD in 2013 and 2017. The GEMM through the Log-Linear (LL) model was used to estimate excess deaths from exposure to ambient PM_2.5_, the formula is shown as (1):

1 $$H{R}_{{\rm{mortality}}}=exp\{\theta f(z)\omega (z)\}$$

where *f*(*z*) = *z* or *f*(*z*) = *log*(*z* + *1*), *HR*_morbidity_ = 1 when *z* = 0 for either form of *f*. Where, *ω*(z) = *1/*(*1* + *exp{−*(*z−µ*)*/*(*τr*)*}*) is a logistic weighting function of *z* and two parameters (*µ, τ*) with *r* the range in the pollutant concentrations. The parameter τ controls the amount of curvature in ω with μ controlling the shape. The set of values of ((*f, µ, τ*) define a shape of the mortality–PM_2.5_ association. The estimation method is based on a routine that selects multiple values of (*f, µ, τ*) and given these values, estimates of θ and its standard error are obtained using standard computer software that fit the Cox proportional hazards model.

#### Relative risk of morbidity

We used the Log-linear function to estimate the long-term morbidity attributable to PM_2.5_ in 2013 and 2017. This function has been applied for several studies in China^15,38^. The cause-specific morbidity relative risk (*RR*_morbidity_) was calculated using the following Eq. (2):

2 $$R{R}_{{\rm{morbidity}}}=\exp \,[\beta \times (C-{C}_{0})]$$

where *β* is the exposure-response coefficients, which means the cause-specific morbidity changes per 1 μg/m^3^ of PM_2.5_ increase; *C* is the annual concentration of PM_2.5_ (μg/m^3^) and *C*_0_ is the reference concentration. We used the reference concentration of PM_2.5_ 10 μg/m^3^ as the morbidity estimation to perform the long-term morbidity assessment, as used in previous studies^15,38^. The *β* coefficient for cause-specific morbidity could be derived from *RR*, using the following Eq. (3):

3 $$\beta =\,\mathrm{ln}(RR)/\Delta C$$

where *RR* can be obtained from the recent epidemiological studies in China; Δ*C* is the change in PM_2.5_ concentration. For example, PM_2.5_ per 10 μg/m^3^ increase, the *RR* for RHA was 1.022 (95% CI: 1.013–1.032)^39^, the *RR* of CHA was 1.013 (95% CI: 1.007–1.019)^40^, the *RR* of CB was 1.029 (95% CI: 1.014–1.044)^39^, the *RR* of AA was 1.021 (95% CI: 1.015–1.028)^41^, and the *RR* of REV was 1.010 (95% CI: 1.005–1.016)^5^, respectively.

### Estimation of health benefits

The health benefits from reduced cause-specific mortality and morbidity were calculated by the difference in health impacts (*HI*) between 2017 and 2013. *HI* was calculated from *HR*_mortality_ and *RR*_morbidity_ for cause-specific disease in 2013 and 2017 using Eq. (4). This function has been applied in previous studies^15^.

4 $$HI=[(RR-1)/RR]\times P\times Pop$$

where *HI* is the health impacts of cause-specific disease; *RR* is the *HR*_mortality_ or *RR*_morbidity_ of cause-specific disease; *P* is the cause-specific mortality or cause-specific morbidity; *Pop* is the population exposed to ambient PM_2.5_.

### Estimation of economic benefits

The associated economic benefits of cause-specific mortality and morbidity were further quantified. The methods used to estimate health economic costs (GDP per capital) of PM_2.5_-related mortality and morbidity in our study followed methodologies in a previously published study^15^, which was based on the willingness to pay (WTP) and cost of illness (COI) methods. The WTP method could be used in calculating the individual willingness to pay for a small reduction of mortality risk and estimating morbidity related economic costs^6,15,42^. The COI method calculated the disease costs in terms of medical treatment, hospitalization, and productivity loss^42^. The health costs for Jinan in 2016 was used and adjusted using GDP per capita from the health costs for Shandong Province in 2016^15^. The health costs (per capita) for Jinan and Shandong Province in 2016 are shown in Table S2.

Lastly, we calculated the total health costs of cause-specific mortality and morbidity in 2013 and 2017 by multiplying the cause-specific *HI* with health costs. The health-related economic benefit was estimated by health costs in 2017 minus that in 2013. The total economic benefit was calculated by adding the benefits from cause-specific mortality and morbidity.

We further projected the co-benefits in health and economics under a future scenario of PM_2.5_ annual concentration reduced from 2013 (110 ug/m^3^) to the national ambient air quality standards Grade-II (15 ug/m^3^)^25^.

All statistical analyses for this study were performed using R software (version 3.2.2, https://mirrors.tuna.tsinghua.edu.cn/CRAN/). Map was created by the packages of *mapdata* (https://cran.r-project.org/web/packages/mapdata/index.html), *maps* (https://cran.r-project.org/web/packages/maps/index.html) and *ggmap* (https://cran.r-project.org/web/packages/ggmap/).

## Results

### Air pollution control policies and implementation

**Figure 3:**
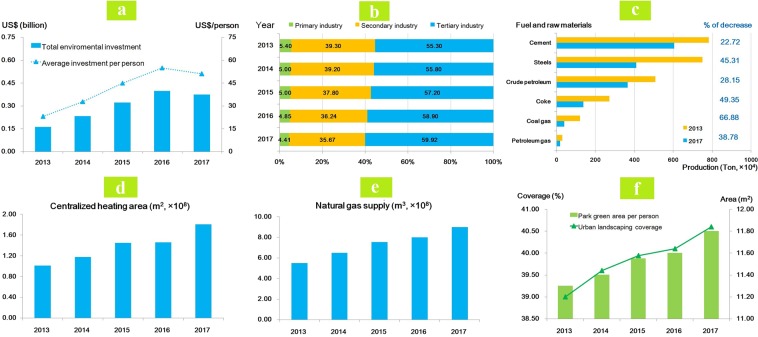
Air pollution control measures in Jinan and directly-related effectiveness, 2013–2017. (**a**) Changes in total environmental investment and average investment per person. (**b**) Changes in adjustment of industrial structure. (**c**) Changes in fuel and raw materials production. (**d**) Changes in centralized-heating area. (**e**) Changes in natural gas supply. (**f**) Changes in urban landscaping coverage (%) and park green area per person (m^2^).

Table S3 summarizes the air pollution control measures and implementation in Jinan between 2013 and 2017. A total of US$1.5 billion was invested on the environmental protection and innovation between 2013 and 2017, with an increasing average annual rate of 25% (Fig. 3a). The average investment was increased from US$13.0 per person in 2013 to US$51.0 per person in 2017.

**Figure 4:**
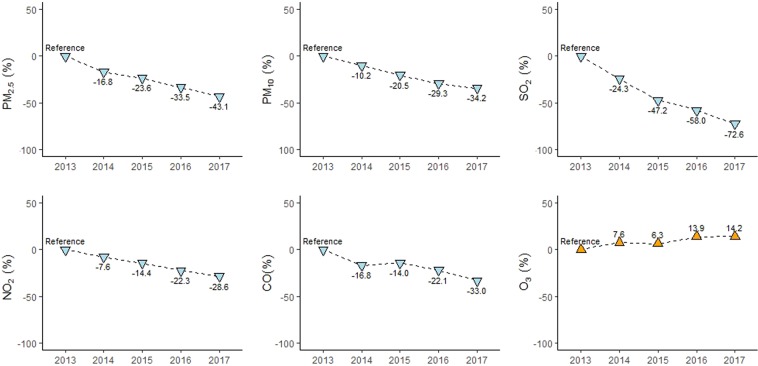
Percent changes (%) in annual mean concentration of air pollutants in Jinan, 2013–2017.

To reduce the emissions from industry and related coal consumption, Jinan had taken the following measures during the study period: (1) Fig. 3b and Table S3 showed the industrial structure adjustment. The proportion of tertiary industry contributed to GDP increased year by year while the proportion of secondary industry totally decreased by 3.63%; (2) Table S3 showed Jinan relocated the old industry around the dense population, reformed the 110 machining enterprises, 53 light enterprises and 7190 small-scale enterprises, and shut down 8 of 9 heavy emissions petrochemical enterprises; (3) reduced the fuel and raw materials production. Compared with 2013, decreased proportions were observed in the production of cement (22.72%), steels (45.31%), crude petroleum (28.15%), coke (49.35%), coal gas (66.88%), and petroleum gas (38.78%) in 2017, respectively (Fig. 3c); (4) among the total 547 coal-fired boilers in Jinan, all 454 coal-fired boilers <35 T/h were eliminated in 2016/17 (Table S3).

To reduce emissions from transportation and vehicle exhaust, the Jinan government provided allowances for the purchase of cleaner vehicles to encourage elimination of Yellow-labeled vehicle after 2013. Further, oil and gas recovery and reformation were taken to all 582 petrol stations in both urban and rural areas (Table S3).

To reduce the emissions from households and related coal consumption, a total of 9,680 households were provided with centralized heating services (Table S3, Fig. 3d). An increase of 0.793 × 10^8^ m^2^ of centralized heating area in 2017 compared with that in 2013. To increase the clean energy (electric and natural gas) supply for households, total natural gas supply reached 9.0 × 10^8^ m^3^ in 2017 with an increasing trend each year (Fig. 3e).

In addition, the Jinan government also improved the urban landscape and green land area, as presented in Fig. 3f. There was a 1.6% increase in urban landscaping coverage. An increasing trend was also observed in green land area.

In order to strengthen environmental air quality monitoring and key air pollution source identification, Jinan government set up the air quality surveillance system with 32 fixed monitoring stations spanning all urban and rural areas by 2016, which helped to achieve a better understanding of the real time distribution of air pollution (Fig. 2).

### Changes of exhaust emissions

We found the city-level exhaust emissions from industry and households dropped significantly, except the soot (dust) emissions from industry (Fig. S2). SO_2_ and nitrogen oxides (NO_x_) emissions from industry showed a greater decrease than that from households between 2013 and 2016. The soot emissions from households presented a 7.67% decrease over the same period.

### Changes in the spatial-temporal profiles of air pollutants

Compared with reference year of 2013, decreased percentages of annual concentration of five air pollutants were observed, from high to low, in SO_2_ (72.6%), PM_2.5_ (43.1%), PM_10_ (34.2%), CO (33.0%), and NO_2_ (28.6%) in 2017, respectively (Fig. 4). In contrast, O_3_ showed a 14.2% increase in 2017. Fig. S3 illustrates the long-term trend of daily air pollutants in Jinan between 2013 and 2017. Except O_3_, the other five air pollutants reached the peak values in January 2013, and then decreased dramatically. Among the 5 air pollutants, SO_2_ presented the most rapidly decreasing trend. O_3_ displayed high concentrations in the warm season and low concentrations in the cold season, which was a different seasonal variation compared with other five air pollutants (Fig. S4).

**Figure 5:**
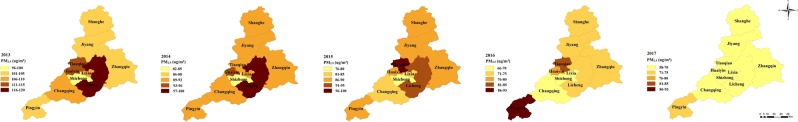
Spatial distribution of ambient PM_2.5_ annual concentration in Jinan, 2013–2017. Map was performed using R software (version 3.2.2, https://mirrors.tuna.tsinghua.edu.cn/CRAN/). The packages of *mapdata* (https://cran.r-project.org/web/packages/mapdata/index.html), *maps* (https://cran.r-project.org/web/packages/maps/index.html) and *ggmap* (https://cran.r-project.org/web/packages/ggmap/) were applied.

Figure 5 showed the spatial distribution of ambient PM_2.5_ between 2013 and 2017. The concentration of PM_2.5_ was extremely high in both urban and rural areas in 2013. Among the ten administrative districts of Jinan, 9 districts had the annual concentration of PM_2.5_ exceed 100 ug/m^3^ in 2013, Licheng district became the most heavily polluted region with a PM_2.5_ annual average value of 120 ug/m^3^, while Shizhong district was featured with the lowest value of 97 ug/m^3^ (Fig. 5, Table S4). In 2017, the annual mean concentration of PM_2.5_ decreased in all ten districts compared with 2013, percentage reduction ranging from 27.88% to 47.50%. PM_2.5_ concentrations showed a faster decreasing trend in urban areas compared to rural areas.

### The health benefits

Results showed decreased burden of cause-specific mortality and morbidity in 2017 attributed to PM_2.5_ in Jinan **(**Table 1**)**. In 2013, a total of 12,591 (95%CI: 8,481–15,349) premature deaths or 33.69% (95%CI: 22.69–41.07%) of the non-accidental deaths, and 37,949 (95%CI: 23349–51183) related cases or 18.76% (95%CI: 11.54–25.30%) of the total morbidity cases were attributed to PM_2.5_. Accordingly, a total of 10,274 (95%CI: 6,728–12,934) premature deaths or 25.21% (95%CI: 16.51–31.74%) of the non-accidental deaths, and 22,129 (95%CI: 13,342–30,465) related cases or 10.46% (95%CI: 6.31–14.40%) of the total morbidity cases were attributed to PM_2.5_ in 2017.

**Table 1:** Disease burden and health benefits associated with ambient PM_2.5_.

Health outcomes	Attributed to PM_2.5_ in 2013	Attributed to PM_2.5_ in 2017	Attributed to PM_2.5_ in future scenario^*^	Health benefits in 2017 compared with 2013	Health benefits in future scenario compared with 2013
**Mortality**					
IHD	4,246 (3,946–4,508)	3,577 (3,282–3,846)	1,704 (1,525–1,877)	669 (621–710)	2,542 (2,362–2,699)
Stroke	6,401 (3,390–8,343)	5,326 (2,679–7,255)	1,601 (722–2,415)	1,075 (569–1,401)	4,800 (2,542–6,256)
LC	617 (419–758)	383 (249–486)	135 (81–185)	234 (158–287)	482 (327–591)
COPD	1,327 (726–1,740)	988 (518–1,347)	361 (173–534)	339 (185–444)	966 (528–1,267)
**Morbidity**					
RHD	7,540 (4,672–10,166)	4,394 (2,669–6,039)	436 (260–613)	3,147 (1,745–4,738)	7,104 (4,021–10,485)
CHD	4,630 (2,575–6,567)	2,645 (1,451–3,802)	257 (139–375)	1,986 (1,025–3,034)	4,373 (2,288–6,591)
CB	12,077 (6,305–17,062)	7,143 (3,609–10,413)	721 (352–1,087)	4,934 (2,143–8,364)	11,356 (5,101–18,641)
AA	12,346 (9,101–15,405)	7,178 (5,222–9,073)	711 (510–912)	5,168 (3,538–6,941)	11,634 (8,074–15,419)
REV	1,356 (696–1,983)	769 (391–1,138)	74 (37–111)	587 (283–913)	1,281 (626–1,972)

Abbreviation: IHD: Ischemic heart disease; LC: lung cancer; COPD: chronic obstructive pulmonary disease; RHA: respiratory disease hospital admission; CHA: cardiovascular disease hospital admission; CB: chronic bronchitis; AA: asthma attack; REV: respiratory disease emergency room visits.

^*^The future scenario was denoted for PM_2.5_ reduced to 15 ug/m^3^.

A total of 2,317 (95%CI: 1,533–2,842) premature deaths were avoided, including 669 (95%CI: 621–710) cases due to IHD, 1,075 (95%CI: 569–1,401) cases due to stroke, 234 (95%CI: 158–287) cases due to LC, and 339 (95%CI: 185–444) cases due to COPD between 2013 and 2017, respectively (Table 1).

Furthermore, in the future scenario of PM_2.5_ reduced to 15 ug/m^3^, a significant amount of mortality could be avoided, including LC mortality was reduced by 78.20% (482 deaths), a reduction of 74.99% (4,800 deaths) in stroke mortality, a reduction of 72.80% (966 deaths) in COPD mortality, and a reduction of 49.87% (2,542 deaths) in IHD mortality.

More than 40% of the morbidities due to RHA, CHA, CB, AA, and REV with an overall of 15,822 (95%CI: 8,734–23,990) cases reduced between 2013 and 2017. Most importantly, nearly 95% of these morbidities could be avoided in the future scenario (PM_2.5_ reduced to 15 μg/m^3^).

### The economic benefits

The related economic benefit from the reduction in mortality and morbidity was presented in Table 2. Total economic benefits of US$ 312.8million (95%CI: 207.0–383.7) associated with reduced mortality and US$ 44.9 million (95%CI: 20.5–74.4) associated with reduced morbidity had been achieved due to decreased PM_2.5_ concentration from 2013 (110 μg/m^3^) to 2017 (63 μg/m^3^). The overall US$ 317.7 million (95%CI: 227.5–458.1) of health economic benefits accounted for 0.29% (95%CI: 0.21–0.42%) of local GDP in 2017.

**Table 2:** Economic benefits associated with reduction in ambient PM_2.5_.

Health outcomes	Health costs in 2013	Health costs in 2017	Health costs in future scenario^*^	Health economic benefits in 2017 compared with 2013	Health economic benefits in future scenario compared with 2013
**Mortality (100 million US$)**					
IHD	5.7 (5.3–6.1)	4.8 (4.4–5.2)	2.3 (2.1–2.5)	0.9 (0.8–1.0)	3.4 (3.2–3.6)
Stroke	8.6 (4.6–11.3)	7.2 (3.6–9.8)	2.2 (1.0–3.3)	1.5 (0.8–1.9)	6.5 (3.4–8.4)
LC	0.8 (0.6–1.0)	0.5 (0.4–0.7)	0.2 (0.1–0.3)	0.3 (0.2–0.4)	0.7 (0.4–0.8)
COPD	1.8 (1.0–2.4)	1.3 (0.7–1.8)	0.5 (0.2–0.7)	0.5 (0.3–0.6)	1.3 (0.7–1.7)
**Morbidity (million US$)**					
RHD	11.6 (7.2–15.7)	6.8 (4.1–9.3)	0.7 (0.4–0.9)	4.9 (2.7–7.3)	11.0 (6.2–16.2)
CHD	10.9 (6.0–15.4)	6.2 (3.4–8.9)	0.6 (0.3–0.9)	4.7 (2.4–7.1)	10.3 (5.4–15.5)
CB	86.5 (45.1–122.2)	51.1 (25.8–74.6)	5.2 (2.5–7.8)	35.3 (15.3–60.0)	81.3 (36.5–133.5)
AA	0.1 (0.1–0.1)	0.1(0.0–0.1)	0.0 (0.0–0.0)	0.0 (0.0–0.1)	0.1 (0.1–0.1)
REV	0.1 (0.1–0.2)	0.1 (0.0–0.1)	0.0 (0.0–0.0)	0.0 (0.0–0.1)	0.1 (0.1–0.2)

Abbreviation: IHD: Ischemic heart disease; LC: lung cancer; COPD: chronic obstructive pulmonary disease; RHA: respiratory disease hospital admission; CHA: cardiovascular disease hospital admission; CB: chronic bronchitis; AA: asthma attack; REV: respiratory disease emergency room visits.

^*^The future scenario was denoted for PM_2.5_ reduced to 15 ug/m^3^.

In the future scenario of PM_2.5_ down to 15 μg/m^3^, a total of US$ 1,289.5 million (95%CI: 825.8–1,673.6) saved from the reduced mortality (US$ 1,186.8 million, 95%CI:777.6–1,460.0) and reduced morbidity (US$102.7 million, 95%CI: 48.2–213.6) could be received, accounting for 1.19% (95%CI: 0.76–1.54%) of GDP in Jinan in 2017.

## Discussion

This is the first study to systematically examine local air pollution measures and quantify environmental, health and economic co-benefits at a city level in China. Our study has analyzed the air pollution control measures and associated co-benefits in a heavily air-polluted city of China between 2013 and 2017, which provides an insight on what and how a single-city could achieve co-benefits of national and local air pollution measures. We have found significant reductions in ambient PM_2.5_, avoided a total of 2,317 premature deaths and 15,822 cases of related diseases, and saved US$ 317.7 million during the study period.

It is important to develop effective air pollution control policies to reduce the production and emissions and to improve public health^4,16^. Coal consumption, for example, became the largest atmospheric pollution source and was identified as the major energy supply in Chinese cities^4,43^. It is clear that coal production, supply and consumption were the core target control factors to the national actions^13,43^. It was estimated that in all types of air pollution sources in China, about 87% of SO_2_ and 67% of NO_x_ were emitted from coal combustion^44^. Our study has observed dramatic changes in coal consumption and reduction in exhaust emissions in Jinan between 2013 and 2017. The percentage of decrease in SO_2_ emissions and NO_x_ emissions from industry was larger in Jinan compared with other heavily air polluted northern cities over the same period, such as Shijiazhuang (51.37% reduction in SO_2_ emissions; 47.07% reduction in NO_x_), Haerbin (60.27% reduction in SO_2_ emissions; 26.21% reduction in NO_x_ emissions)^35^. In early 2018, Jinan government further issued “the Jinan Working Program to Reduce Coal Consumption from 2018 to 2020”, which required coal consumption reductions from households^45^. Our study proves that these measures in Jinan are effective in controlling air pollution.

Previous national studies have shown improved air quality in China due to the APPCAP^4,14^. Compared with national studies, the achieved reduction of air pollution in Jinan is above the national average. The 25% reduction in PM_2.5_ in Jinan has met the Ten Specific Tasks of APPCAP^13^. The WHO 2018 report on global air pollution indicates that about 91% population live in areas with an annual average PM_2.5_ > 10 ug/m^3^^3,10^. We have found that nearly 7 million people exposed to severe air pollution with ambient PM_2.5_ > 100 ug/m^3^ in Jinan in 2013. Due to air pollution controls and implementation, all ten districts of Jinan have achieved consistent reduction in ambient PM_2.5_ concentration by 2017, with more benefits in urban areas. However, the annual concentration of PM_2.5_ in all districts of Jinan is still significantly higher than the national ambient air quality standards Grade-I (35 ug/m^3^)^25^, which indicates further air pollution policies and measures would be necessary for Jinan. We have also noted the significant increase in O_3_ during study period, which is consistent with other national studies^14^. It indicates that O_3_ and other air pollutants still pose an ongoing challenge to the health of people in Jinan and other similar cities in China.

WHO recently reported that the ambient air pollution was the leading cause of the mortality due to the chronic diseases^10^. Our results indicate that premature deaths attributed to PM_2.5_ exposure were three times larger than that at national average level^16^. After the implementation of the air pollution controls, Jinan has achieved a 50% reduction in PM_2.5_ and saved a total of 18.4% premature deaths in 2017. It is worthy to note that appreciable health benefits could be reduced if PM_2.5_ further reduces to 15 μg/m^3^. Additionally, significant decrease in morbidity can be achieved as suggested in our historical and scenario-based modeling analyses. Among the five cause-specific diseases, acute bronchitis and asthma attacks affected most people, which generally agreed with previous studies^15^.

Adverse health outcomes attributed to PM_2.5_ exposure could lead to tremendous economic burdens^6,15,16^. Our results support this statement and indicate a reduction in GDP between 2013 and 2017. Our finding is consistent with previous study in the central area of China^16^. The average economic loss ranged from 0.3% to 1.0% of the total GDP of 190 Chinese cities from 2014 to 2016^16^. Our estimation demonstrates higher economic burdens than that at the national level because of the heavy air pollution in Jinan^16^. Another reason is that our estimation of the economic burdens included both mortality and morbidity. Not surprising, Jinan has achieved more economic benefits due to improvement in air quality and lives saved. We have also reported more economic burden due to premature deaths (94.0% in 2013 and 95.6% in 2017), which is similar to the finding of Yin *et al*.^38^ who reported more than 80% of the overall external costs due to premature deaths in Beijing. The findings imply that we need to pay more attention to primary health care to prevent premature deaths from air pollution.

Limitations of the study should be acknowledged. First, due to data unavailability, the usage of national morbidity data as a proxy of Jinan population was not ideal. Second, the air monitoring stations did not cover the rural areas of Jinan before 2016, which could affect the spatial analysis of air pollution in Jinan before 2016. Last, in the projection of the benefits under the future scenario of PM_2.5_ down to 15 μg/m^3^, some other factors that change with time, e.g. demographic data, socio-economic situation and climate change adaptation, could not be included in the analysis. Finally, the emission reduction estimated is not directly linked with policies because the secondary chemical formation also contributed an important fraction.

## Conclusion

Our study provides evidence of the effectiveness and co-benefits of local air pollution control measures in the study city of China. Findings suggest that government efforts to improve air quality will lead to significant environmental, health and economic benefits. Current policies should continue to achieve better air quality standards in order to prevent more premature deaths, morbidities and economic losses. Lessons learned from the study city could be applicable to other cities and regions with severe air pollution in China and globally.

## Supplementary information

Supplemental files.
