# Supplementary material for: Analyses of air pollution control measures and co-benefits in the heavily air-polluted Jinan city of China, 2013–2017
Source: Sci Rep. 2020 Mar 25;10:5423. doi: 10.1038/s41598-020-62475-0 (PMC7096483; doi:10.1038/s41598-020-62475-0)
Supplement: Supplementary file 1 — Supplemental files. [file 41598_2020_62475_MOESM1_ESM.docx]

**Supplemental files**

**Analyses of air pollution control measures and co-benefits in a heavily air-polluted Jinan city of China，2013-2017**

Liangliang Cui^a,b^, JingwenZhou^a^, XiumiaoPeng^a^, Shiman Ruan^a,*^, Ying Zhang^b,*^

^a^ *Department of Environmental Health, Jinan Municipal Center for Disease Control and Prevention, Jinan City, Shandong Province 250021, China*

^b^*The Sydney School of Public Health, University of Sydney, NSW, 2006, Sydney, Australia*

^*^**Corresponding authors**: Address correspondence to Ying Zhang, The Sydney School of Public Health, University of Sydney. Address: Room 333, Edward Ford Building A27, NSW, 2006, Sydney, Australia. E-mail: [ying.zhang@sydney.edu.au](mailto:ying.zhang@sydney.edu.au). Please also correspondence to Shiman Ruan, Jinan Municipal Center for Disease Control and Prevention. Address: Room 704, NO. 2 Weiliu Road, Huaiyin District, Jinan City, Shandong Province 250021, China. E-mail:[shimanruan@qq.com](mailto:shimanruan@qq.com).


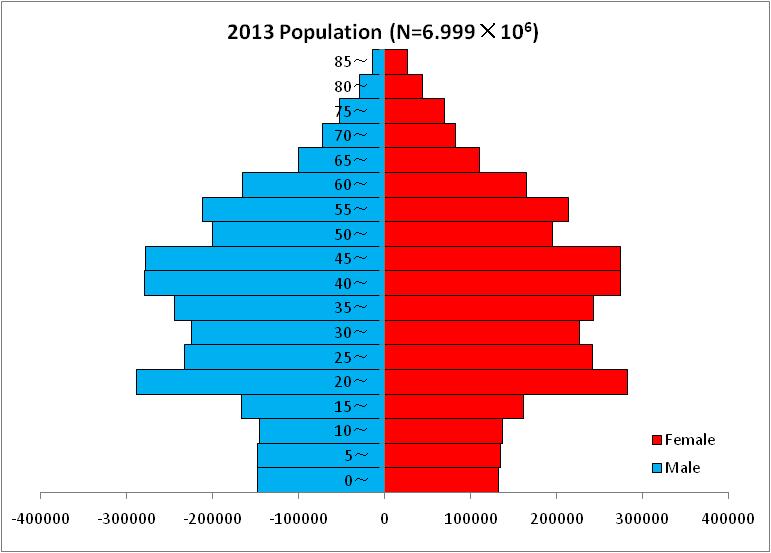

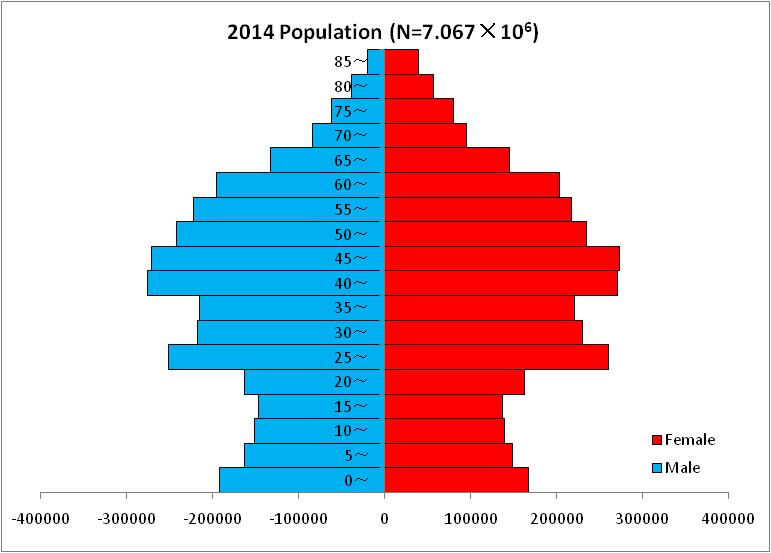


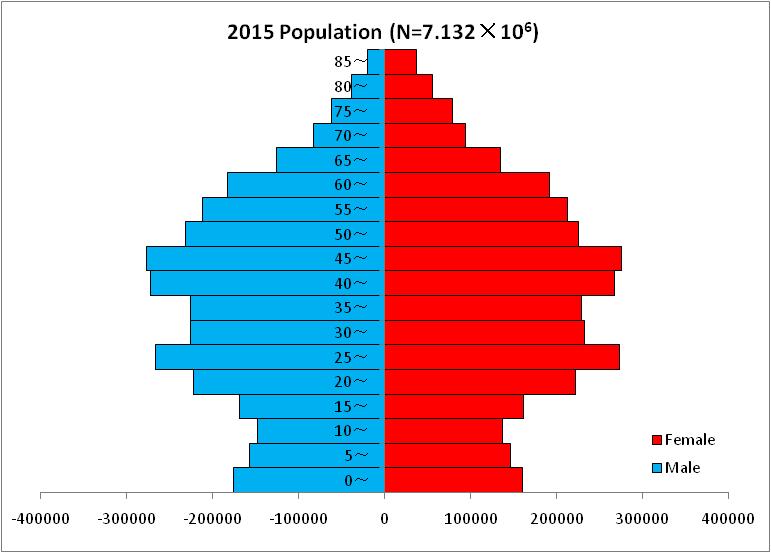

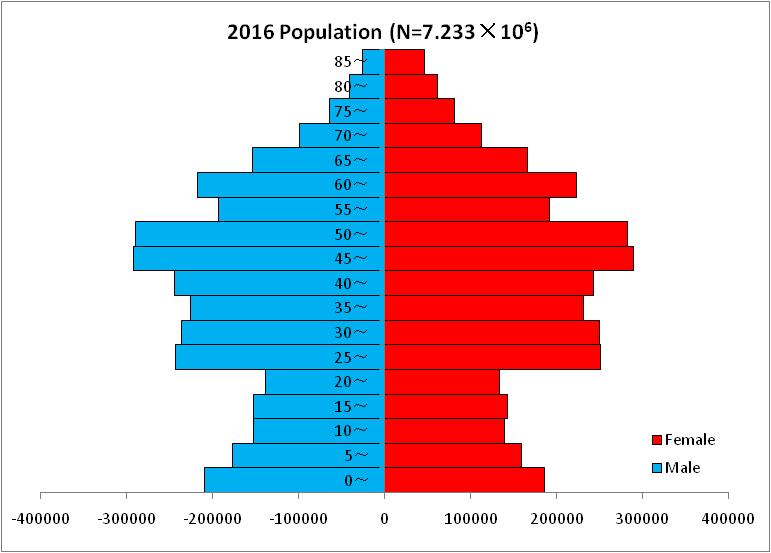


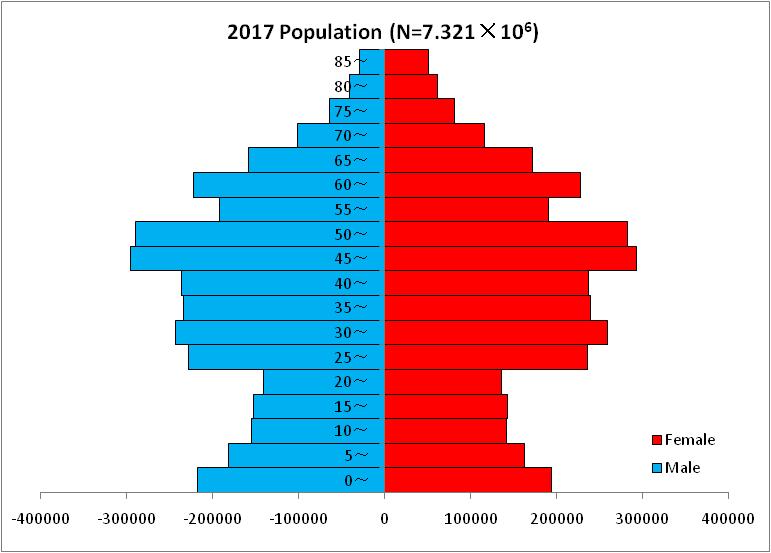


**Fig. S1.** Population pyramidsby age and gender in Jinan, 2013-2017

**Table S1.**Population, all-cause mortality and [gross](javascript:void(0);) domestic [product](javascript:void(0);) (GDP) in Jinan, 2013-2017

| Year | Population (×10^6^) | All-cause mortality (‰) | GDP (billion RMB) | GDP (billion US$) |
| --- | --- | --- | --- | --- |
| 2013 | 6.999 | 6.82 | 523.019 | 78.741 |
| 2014 | 7.067 | 6.84 | 577.060 | 86.877 |
| 2015 | 7.132 | 6.67 | 610.023 | 91.839 |
| 2016 | 7.233 | 6.76 | 653.612 | 98.401 |
| 2017 | 7.321 | 6.91 | 720.196 | 108.426 |

**Table S2.**Economic costs (US$) per capital of cause-specific health outcomes in Jinan and Shandong Province

| Health outcomes | Shandong Province | Jinan city |
| --- | --- | --- |
| All-cause mortality | 102182.5 | 135020.2 |
| Respiratory disease hospital admission | 1167.7 | 1543.0 |
| Cardiovascular disease hospital admission | 1776.0 | 2346.7 |
| Chronic bronchitis | 5418.8 | 7160.2 |
| Asthma attack | 5.4 | 7.1 |
| Emergency room visits | 62.3 | 82.3 |

**Table S3.** Air pollution control measures in Jinan, 2013 – 2017.

| Strategies and policies | Goals and measures |
| --- | --- |
| Increase the investment on the environmental protection and innovation | - Total of US$1.48 billion were invested on the environmental protection and innovation during 2013-2017. - An average annual increasing rate was 105% compared with 2013 (US$0.16 billion). |
| Adjustment of industrial structure | - Keep the key role of tertiary industry and the proportion of tertiary industry in GDP increased 4.62% in 2017 compared with 2013. - Decrease the proportion of secondary industry in GDP with a 3.63% reduction in 2017 compared with 2013. |
| Industry relocation, reformation and shut down | - 8 of 9 petrochemical enterprises were closed. - 110 machining enterprises were reformed. - 53 light industry enterprises were reformed. - 7190 small scale enterprises were shut down and reformed. |
| Reduce the fuel and raw materials production | - Cement production decreased 22.72% in 2017 compared with 2013. - Steels production decreased 45.31% in 2017 compared with 2013. - Crude petroleum production decreased 28.15 in 2017 compared with 2013. - Coke production decreased 49.35% in 2017 compared with 2013. - Coal gas production decreased 66.88% in 2017 compared with 2013. - Petroleum gas production decreased 38.78% in 2017 compared with 2013. |
| Eliminate small coal-fired boilers | - 83% (454/547) of coal-fired boilers has been eliminated by August 2017, including 131 coal-fired boilers < 35 T/h in 2016 and 323 coal-fired boilers < 35 T/h in 2017, which means there was no coal-fired boilers < 35 T/h in Jinan. |
| Yellow-labeled vehicle elimination and oil reformation | - Provide allowance to encourage the Yellow-labeled vehicle elimination. - All of 582 petrol stations both in urban and rural areas were provided oil and gas recovery and reformation. |
| Develop centralized heating and decrease the household coal consumption | - Expand centralized heating covering: a total of 9680 households (4940 households in 2016 and 4740 households in 2017) were provided with centralized heating. - Centralized heating area had increased 0.793×10^8^ m^2^ from 2013 (1.013×10^8^ m^2^) to 2017 (1.806×10^8^ m^2^). |
| Increase clean energy supply | - A total of 1.1×10^5^ households were supplied with clean energy (electric and natural gas) instead of coal burning. - 6.0×10^5^ households were converted to cleaner fuels. - Nature gas supply raised to 9.0×10^8^ m^3^ in 2017, increased 63.64% compared with that in 2013 (5.5×10^8^ m^3^). |
| Increase urban landscaping | - The urban landscapingcoverage reached to 40.60% in 2017 with a 1.60%increased compared with 2013. - The green land area per person increased 0.5 m^2^ in total. |
| Increase air quality fixed monitoring stations | - The number of fixed monitoring stations reached to 15 in 2013 since the first three fixed monitoring stations were set up in 2011. - A total of 32 fixed monitoring stations were set up in 2016, which covered whole urban and rural areas of Jinan. |

**
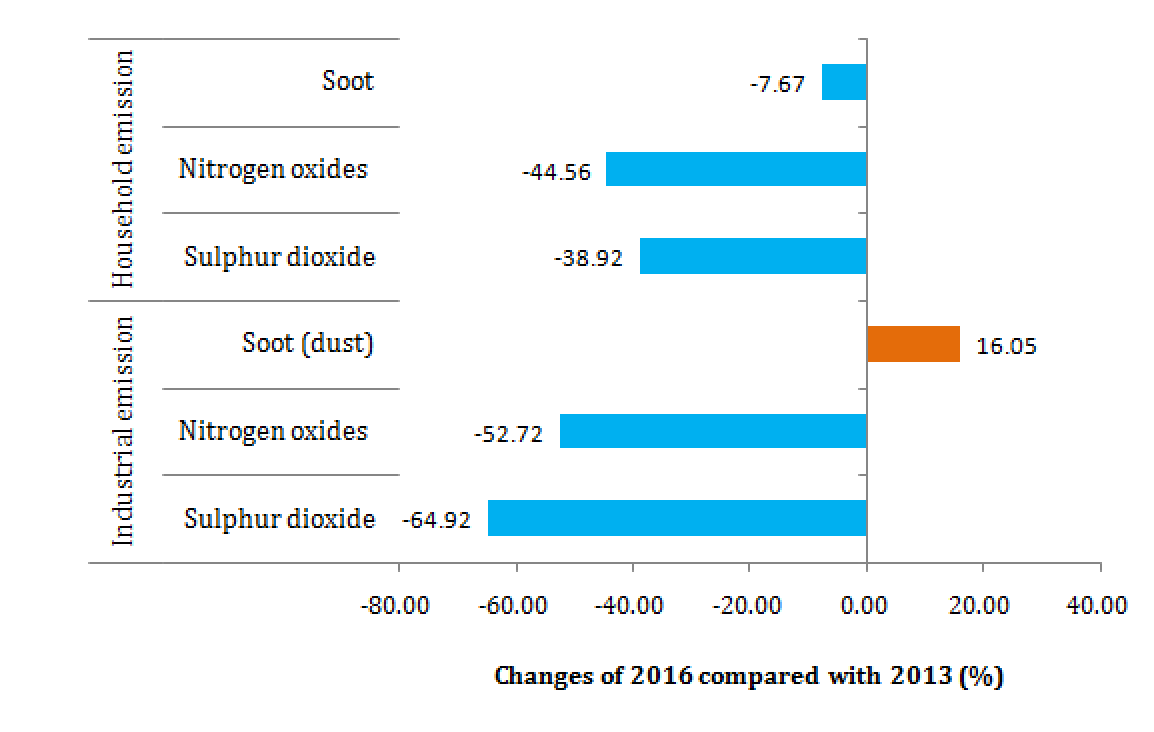
**

**Fig. S2.**Exhaust emission from industry and household in 2016, compared with 2013 in Jinan.

**
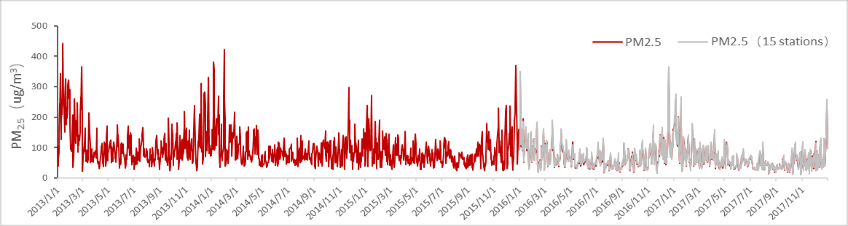
**

**
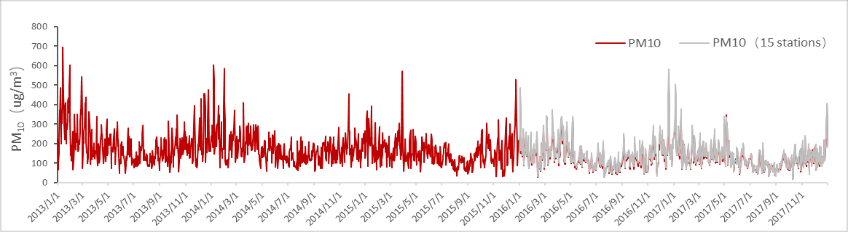
**

**
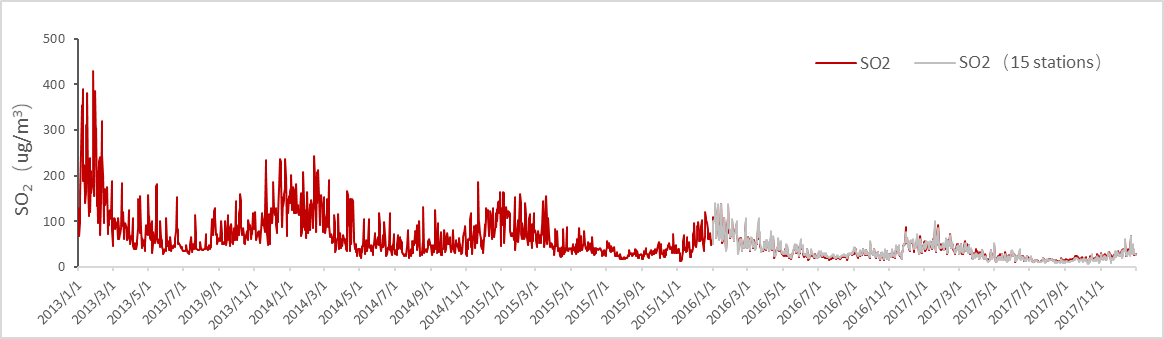
**

**
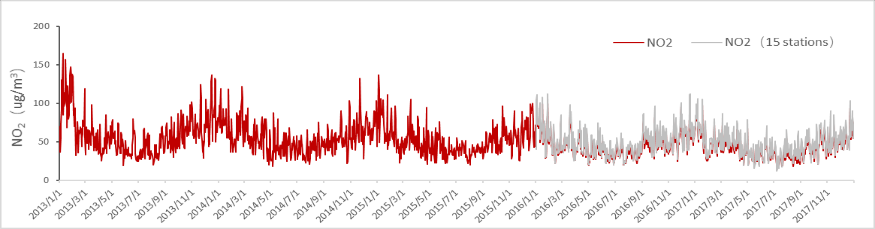
**

**
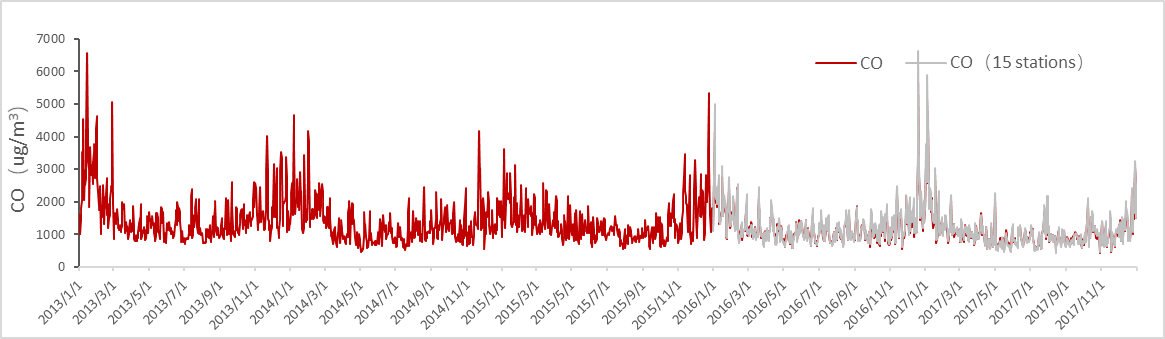
**

**
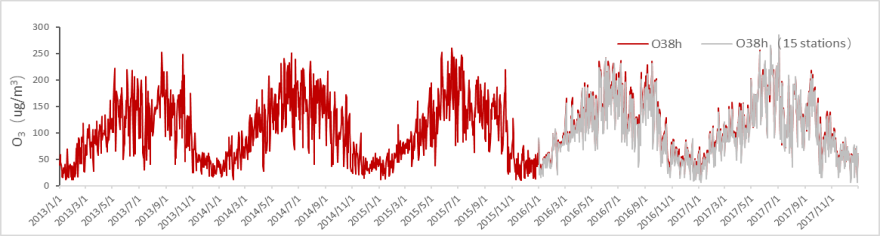
**

**Fig. S3.**Long-term trend of daily air pollutants in Jinan, 2013-2017.

Note: Light grey line denoted for the daily average concentration of air pollutants calculated from 15 fixed air monitoring stations set up before 2016. Pearson Correlation Coefficient of daily average air pollutants between 15 stations and 32 stations during 2016 to 2017 was: 0.993 for PM_2.5_, 0.992 for PM_10_, 0.985 for SO_2_, 0.985 for NO_2_, 0.991 for CO, 0.996 for O_3_.

**
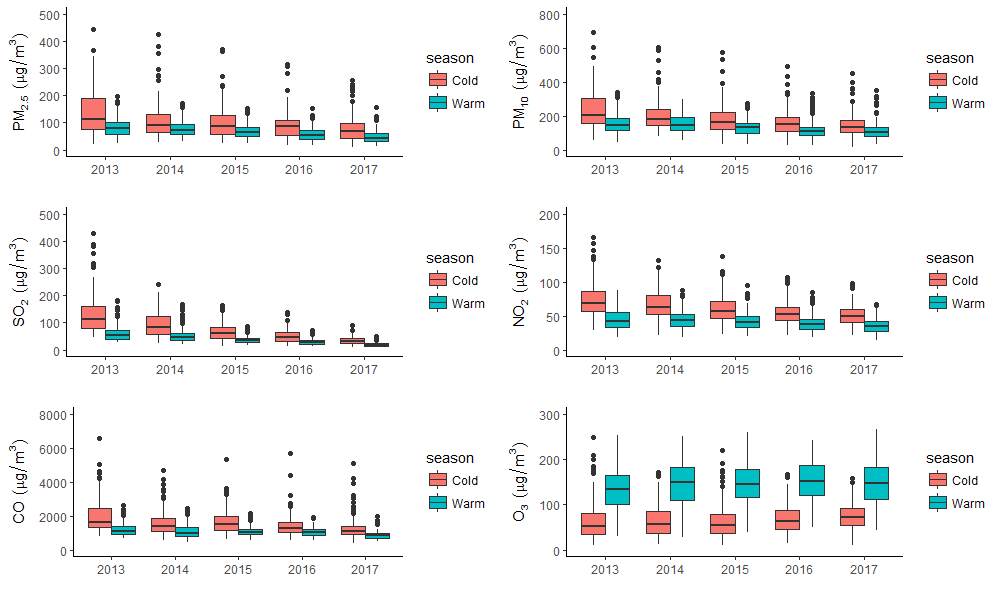
**

**Fig. S4.**Season-trend of air pollutants in Jinan, 2013-2017.

**Table S4.** Percent changes (%) in ambient PM_2.5_ concentration in all ten districts of Jinan in 2017 compared with 2013.

| District | Region | 2013 (ug/m^3^) | 2017 (ug/m^3^) | Changes (%) of 2017 compared with 2013 |
| --- | --- | --- | --- | --- |
| Licheng | Urban | 120 | 63 | -47.50 |
| Huaiyin | Urban | 113 | 63 | -44.25 |
| Lixia | Urban | 107 | 61 | -42.99 |
| Shizhong | Urban | 97 | 58 | -40.21 |
| Tiaoqiao | Urban | 113 | 68 | -39.82 |
| Changqing | Rural | 108 | 60 | -44.44 |
| Shanghe | Rural | 104 | 63 | -39.42 |
| Jiyang | Rural | 104 | 65 | -37.50 |
| Zhangqiu | Rural | 104 | 67 | -35.58 |
| Pingyin | Rural | 104 | 75 | -27.88 |
